# Calcific Aortic Valve Disease-Natural History and Future Therapeutic Strategies

**DOI:** 10.3389/fphar.2020.00685

**Published:** 2020-05-13

**Authors:** Brunilda Alushi, Lavinia Curini, Mary Roxana Christopher, Herko Grubitzch, Ulf Landmesser, Amedeo Amedei, Alexander Lauten

**Affiliations:** ^1^Department of Cardiology, Charite´ Universitätsmedizin Berlin and German Centre for Cardiovascular Research (DZHK), Berlin, Germany; ^2^Department of General and Interventional Cardiology, Helios Klinikum Erfurt, Erfurt, Germany; ^3^Department of Experimental and Clinical Medicine, University of Florence, Firenze, Italy; ^4^Berlin Institute of Health, Berlin, Germany; ^5^Department of Cardiology, German Heart Centre Berlin (DHZB), Berlin, Germany; ^6^Sod of Interdisciplinary Internal Medicine, Azienda Ospedaliera Universitaria Careggi (AOUC), Florence, Italy

**Keywords:** severe aortic stenosis, calcific aortic valve, aortic valve replacement, TAVR, microbiome, immune system, inflammation

## Abstract

Calcific aortic valve disease (CAVD) is the most frequent heart valve disorder. It is characterized by an active remodeling process accompanied with valve mineralization, that results in a progressive aortic valve narrowing, significant restriction of the valvular area, and impairment of blood flow.The pathophysiology of CAVD is a multifaceted process, involving genetic factors, chronic inflammation, lipid deposition, and valve mineralization. Mineralization is strictly related to the inflammatory process in which both, innate, and adaptive immunity are involved. The underlying pathophysiological pathways that go from inflammation to calcification and, finally lead to severe stenosis, remain, however, incompletely understood. Histopathological studies are limited to patients with severe CAVD and no samples are available for longitudinal studies of disease progression. Therefore, alternative routes should be explored to investigate the pathogenesis and progression of CAVD.Recently, increasing evidence suggests that epigenetic markers such as non-coding RNAs are implicated in the landscape of phenotypical changes occurring in CAVD. Furthermore, the microbiome, an essential player in several diseases, including the cardiovascular ones, has recently been linked to the inflammation process occurring in CAVD. In the present review, we analyze and discuss the CAVD pathophysiology and future therapeutic strategies, focusing on the real and putative role of inflammation, calcification, and microbiome.

## Introduction

Calcific aortic valve disease (CAVD) is the most common valve disease worldwide (Nkomo et al., 2006). Epidemiological studies show that 2.8% of adults over 75 years old have some CAVD degree, and as many as 25% of adults over 65 years old have at least valvular sclerosis (Miller et al., 2011).

CAVD is a chronic process characterized by progressive fibrotic tissue remodeling and mineralization (Mathieu et al., 2015). Over the years, there is a disease continuum from sclerosis to chronic inflammation and finally leaflet calcification, culminating with severe stenosis. Human pathologic samples have shown that key features in the CAVD development include pathological concentrations of inflammatory cells and lipid species (Otto et al., 1994; O’Brien et al., 1996).

Several risk factors have been identified as relevant in the CAVD progression. Among others, male gender, high triglyceride levels, and smoking have been independently associated with early aortic valve replacement in the presence of CAVD. Levels of oxidative stress, higher in patients that are more exposed to certain risk factors than others, could be responsible for the starting of the sclerotic CAVD phase (O’Brien, 2006; Ferreira-Gonzalez et al., 2013).

Calcification is recognized as an active disease process driven by the native valvular interstitial cells (VICs) (Sun et al., 2013). These cells acquire an osteogenic and pro-calcific profile in response to different pathological stimuli, such as inflammatory mediators, endothelial damage, low-density lipoprotein (LDL) accumulation, reactive oxygen species (ROS), increase calcium/phosphate (Ca/Pi) levels, modified lipids, and cyclic stretch (Liao et al., 2008; Rajamannan et al., 2011; Rattazzi et al., 2014). The underlying process that ends with the ectopic mineralization of the aortic valve is still not entirely understood. Treatment options include operative valve replacement and percutaneous implantation of valve prosthesis (Lauten et al., 2013; Lauten et al., 2014; Daubert et al., 2016; Alushi et al., 2019; Wernly et al., 2019).

To date, there is no medical treatment available to prevent or reverse calcium deposition within the valve leaflets. Conventional cardiovascular drugs studied in clinical trials failed to influence the disease progression or reduce adverse outcomes, therefore additional research is required to understand the mechanisms of disease progression and, identify novel therapeutic targets (Cowell et al., 2005; Freeman and Otto, 2005; Rossebo et al., 2008; Chan et al., 2010).

## CAVD Biology

### Histological Structure of the Aortic Valve

**Figure 1:**
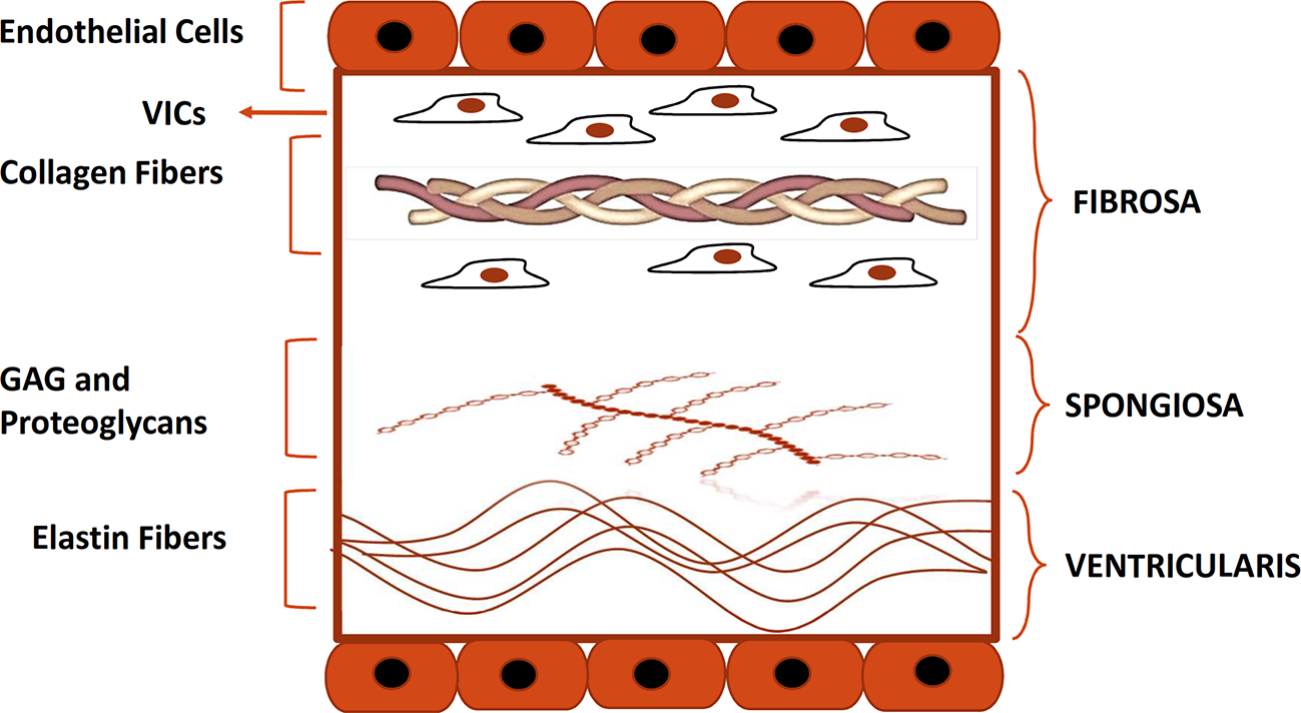
Histological structure of the aortic valve. Depicted is the histological structure of the healthy aortic valve. Fibrosa, spongiosa, and, ventricularis are the three layers that make up the structure of a normal aortic valve. The Fibrosa layer is composed of type I and III collagen fibers and contains also VICs. Spongiosa and ventricularis layers are respectively composed of GAG and proteoglycans and elastin fibers. Endothelial cells form a monolayer on each side of the cusp. GAG, glycosaminoglycans; VIC, valve interstitial cell.

The aortic heart valve consists of three leaflets that allow blood flow from the left ventricle to the aorta without regurgitation (Liao et al., 2008). Each leaflet has a trilaminar structure that is vital for the biomechanical properties of the aortic valve (Tseng and Grande-Allen, 2011; Lindman et al., 2016) as shown in **Figure 1**. Histologically, the leaflets are composed of three distinct layers: fibrosa, spongiosa, and ventricularis. Fibrosa and ventricularis are the external layers, facing the aorta and the left ventricle respectively. Fibrosa consists largely of collagen fibers with dispersed VICs, that are thought to be responsible for reinforcing the valvular structure (Dweck et al., 2012a).

The central layer, spongiosa, is rich in glycosaminoglycans (GAGs) and is responsible for absorbing some of the mechanical stress generated during the cardiac cycle.

The ventricularis is localized on the ventricular side of the leaflets and consists of collagen and elastin fibers (Chen and Simmons, 2011; Mathieu et al., 2015). The tissue composition of ventricularis provides more compliance and grants the apposition of free edge leaflet regions, thus preventing the backward blood flow into the left ventricle during diastole (Lindman et al., 2016). The normal valve leaflet is avascular and free of infiltrating lymphocytes or monocytes.

### Pathobiology of CAVD

**Figure 2:**
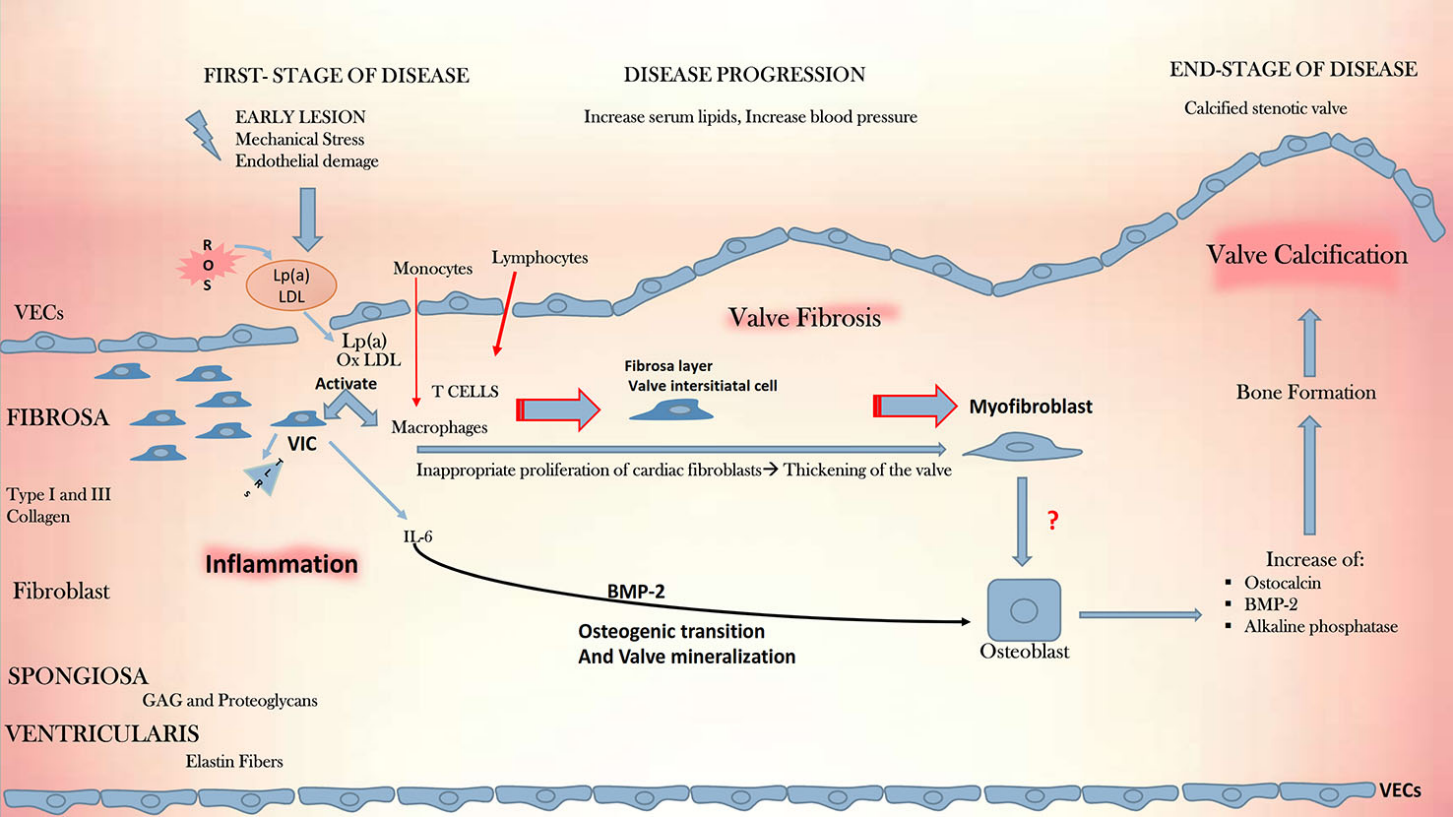
CAVD development. Depicted is the pathophysiologic cascade leading to CAVD. Initial lesions, such as mechanical stress, endothelial damage and, production of ROS, initiate an ox-LDL-mediated inflammation in the endothelium and promote the activation of macrophages and quiescent VICs. These start to express TLRs and the cytokine pro -inflammatory IL-6, which plays a fundamental role in inflammation, together with macrophages and T cells. IL-6 mediates mineralization through the BMP-2 signal, driving the osteogenic transition and culminating with the aortic valve calcification. CAVD, calcific aortic valve disease; GAG, glycosaminoglycans; IL-6, interleukin 6; Lp(a), lipoprotein a; ox-LDL, oxidized low-density lipoprotein; ROS, reactive oxygen species; TLRs, toll-like receptors, VECs, valve endothelial cells; VICs, vascular interstitial cells, BMP-2, bone morphogenetic protein 2.

The CAVD pathophysiology is schematically depicted in **Figure 2**. The process of progressive fibrocalcific remodeling of the aortic valve is multifactorial, involving genetic predisposition, endothelial shear stress, chronic inflammation, lipid deposition, and valve calcification (Freeman and Otto, 2005; Perrot et al., 2019; Thériault et al., 2019). In its early stages, CAVD resembles atherosclerosis. The existence of shared risk factors for the disease development and the correlation between the severity of CAVD and that of coronary calcification suggest a shared disease pathway, at least in the initial phases of both diseases.

Alike atherosclerosis, the triggering event in CAVD is endothelial damage resulting from increased mechanical stress and reduced shear stress. Physiologic fluid shear stress (FSS) contributes to valve homeostasis, whereas altered shear stress, on the contrary, stimulates the endothelial upregulation of vascular cell adhesion molecule-1 (VCAM-1) and intracellular adhesion molecule-1 (ICAM-1) (Sucosky et al., 2009; Sun et al., 2013). The endothelial damage following the altered shear stress allows the infiltration of lipids, especially LDL and lipoprotein (a) [Lp(a)], starting the recruitment of inflammatory cells into the leaflets. Later appears the formation of focal subendothelial plaque-like lesions constituted from LDL, Lp(a) and infiltrates of inflammatory cells which interact in processes that release ROS causing LDL oxidation. In time, the expansion of this inflammatory process triggers the release of factors that cause VICs to acquire an osteogenic profile, with the formation of microcalcifications initiating leaflet mineralization (Otto et al., 1994; O’Brien et al., 1996). The two disease processes differ in the later disease phases: while in atherosclerosis, smooth muscle cells are the active players in the chronic inflammation within the plaque, in CAVD fibroblasts are involved in the prominent mineralization process and only with a small number of smooth muscle cells is present.

The hallmark of the early CAVD stages is inflammation, characterized by the activation of the leaflet endothelium *via* enhanced expression of cell adhesion molecules (VCAM-1, ICAM-1) (Sun et al., 2013; de Sousa et al., 2017). Disease initiation involves the activation of VICs, recruitment of immune cells, and subsequent sclerosis of the valve leaflets owing to fibrosis and formation of calcific nodules. All these phenomena start and interest more the fibrosa layer within the valve. The first macroscopic change in the leaflets, seen as microcalcifications, or focal thickening with preserved valve function, is nominated aortic valve sclerosis, nonetheless, the initiating events likely occur much earlier. The activation degree of the immune system seems to be different on different examined valve areas. Interestingly, the thickening process and the formation of calcium nodules accompanied by neo-angiogenesis, are both localized near the aortic surface of the leaflets (Cote et al., 2013).

The final disease, namely calcific aortic stenosis, is characterized by large calcified noduli on the leaflet surface, that protrude into the sinuses of Valsalva, hindering the leaflet mobility (Rajamannan et al., 2011). In this final phase the leaflets are infiltrated by immune cells and concomitantly angiogenesis occurs, along with deposition of lipids, proteoglycans, and cell debris (Chen and Simmons, 2011). Finally, the calcification of the valvular matrix leads to an increased stiffness and obstruction of the blood flow (**Figure 2**). With an orifice under 1 cm^2^, versus 2.5–4.5 cm^2^ observed in a normal valve, the stenotic valve generates a pressure gradient of over 40 mmHg, categorized as severe stenosis with an indication to valve replacement (Zigelman and Edelstein, 2009; Rutkovskiy et al., 2017).

#### Impact of Inflammation in CAVD Remodeling

Inflammation is the primary response of innate immunity and occurs after endothelial damage with its activation and lipid deposition. The innate immune response, with both its components, cellular and humoral responses are implicated in this process. As response to an injury induced by foreign organisms, dead cells or physical irritants, the innate immune system represents the first response to external or internal triggers and initiates the process of tissue regeneration (Hulin et al., 2018; De Almeida et al., 2019). Once the inflammation process has been triggered, it will proceed along a certain course of events until the inflammatory stimulus is eradicated and the healing mechanism can begin. However, if the inflammatory source cannot be eliminated, inflammation will progress, varying in intensity over time (Hakansson and Molin, 2011).

Histological samples of human CAVD valves are characterized by calcified areas rich in lymphocytes, macrophages, and osteoblast-like cells (Hulin et al., 2018). The inflammatory process can be acute or chronic (Pahwa and Jialal, 2019). Cote´ et al. showed that chronic inflammatory infiltrates, composed of the CD45^+^ leukocytes, CD68^+^ macrophages, and a few scattered CD3^+^ T cells, were present near the calcified areas. Moreover, the chronic inflammatory infiltrates in the aortic valve were independently associated with several indices of remodeling, suggesting that inflammation may participate in mineralization and the fibrotic process (Cote et al., 2013). A significant association between the degree of aortic valve inflammation and the development of calcification has been previously reported (and recently confirmed) using 18F-fluorodeoxyglucose positron emission tomography. In these studies, a high degree of inflammation and calcification was documented in patients with severe CAVD, with the latter being the predominant pathogenic process (Marincheva-Savcheva et al., 2011; New and Aikawa, 2011; Dweck et al., 2012b). This could in part explain the failure of statin therapy in slowing the CAVD progression (Cowell et al., 2005; Rossebo et al., 2008).

Previously thought to be a passive disorder, CAVD is now recognized as an active process, whereby endothelial progenitor cells (EPCs) and inflammatory cells promote tissue remodeling (Yip and Simmons, 2011). In summary, mechanical shear stress and atherogenic factors activate VICs and initiate the recruitment of inflammatory cells. The following remodeling of extracellular matrix with leaflet stiffening and valvular dysfunction causes further mechanical stress that maintains the self-perpetuating cycle of endothelial dysfunction. As suggested by studies with molecular imaging, the continuous maintenance of this shear stress-inflammation cycle could result irreversibly to calcium deposition and finally to severe CAVD (New and Aikawa, 2011).

##### Role of Lipids

Over recent years, several studies have highlighted the central role of ox-LDL as an activator of inflammation, both in atherosclerosis and in CAVD (Cote et al., 2008). In atherosclerotic plaques, ox-LDL activates the inflammatory cells and the production of cytokines promoting tissue remodeling and disease progression (Hansson, 2005). Existing evidence of valve infiltration by ox-LDL in cases of CAVD, supports the concept that the development of valve calcification may be, at least in part, influenced by ox-LDL and, that an association exists between high plasma levels of ox-LDL and CAVD (Mohty et al., 2008). Although having some similarities with atherosclerosis, CAVD differs from it since the aortic valve has some inherent properties that differ from the vascular wall. Indeed, the cellular organization, as well as the hemodynamic stress imposed upon the aortic valve, vary from that of the arteries. Furthermore, the phases that characterize the pathological process occurring within the valve, from early inflammation stages to the calcification ones, are more numerous and qualitatively different from atherosclerosis, as shown in **Figure 2**. Most importantly, statins, although efficient in reducing adverse events in patients with atherosclerosis, are inefficient in CAVD (Cowell et al., 2005; Rossebo et al., 2008; Chan et al., 2010).

#### Role of the Immune Response in CAVD

The immune system, with innate and adaptive responses, plays a central role in the development of different chronic disorders, including atherosclerosis and CAVD. Evidence suggests that inflammation, as the primary response of the innate immunity, promotes the mineralization of VICs in response to several factors. Adaptive immunity, on the other hand, could play a role in instrumenting the immune response (Cote et al., 2013). In this regard, experimental studies have shown that the increased leukocyte density in mineralized aortic valves correlates with faster disease progression (Mathieu et al., 2015).

##### Innate Response in CAVD

The innate immune response in CAVD |+|^}_–^be initially triggered by several oxidized lipid species, through both the toll-like receptors (TLRs) and the nuclear factor-κ B (NF-κB) pathway. VICs express TLRs, known to play a key role in inflammation and initiation of antigen-specific adaptive immune responses. TLRs can be activated by several lipid species, especially ox-LDL, with the signal passing through the recruitment of specific adaptor molecules to the activation of the transcription factor NF-κB (Kawasaki and Kawai, 2014; Garcia-Rodriguez et al., 2018). In this way, TLRs signal inflammation through the NF-κB pathway and are vital for maintaining tissue homeostasis.

###### NF-Kappa B Pathway

The NF-κB plays an important role in signal integration by responding to mediators of endothelial injury. VICs actively participate in the regulation of inflammation by producing a high level of cytokines, and the NF-κB pathway contributes to a stimulus-dependent and cell-type-specific manner. The NF-κB activation (canonical and non-canonical) pathway is mediated by different extracellular signals including angiotensin II, ox-LDL, CD40 ligand, advanced glycation end-products, and inflammatory cytokines. The canonic pathway is activated, among others, by tumor necrosis factor alpha (TNF-α) and interleukin-1β (IL-1β). Angiotensin II-induced ROS generated also on mineralized aortic valves, are involved in the activation of the NF-κB canonic pathway (Jamaluddin et al., 2007). The non-canonical pathway is activated, among others, by vasoactive peptides, ox-LDL, activated CD40 receptor, B cell-activating factor (BAFF), lymphotoxin β (LTβ) monocyte released cytokines, or advanced glycation end-products (Xiao et al., 2004; Morrison et al., 2005). In summary, activated NF-κB controls the initiation of vascular inflammation, through “leucocyte adherence” and “chemotaxis” and is a crucial signaling integrator of vascular injury.

###### Cytokines

One important target of the NF-kB pathway is IL-6. Highly expressed in valves with severe CAVD, IL-6 is a pleiotropic cytokine secreted by multiple vascular cell types, such as macrophages, lymphocytes, fibroblasts, endothelial cells, and smooth muscle cells. It mediates local vascular monocyte activation and protection from ROS-induced cellular stress *via* the downstream transcription effector signal transducer and activator of transcription 3 (STAT3) (Brasier, 2010; El Husseini et al., 2014; Rahat et al., 2016; Huang et al., 2019). By controlling the monocyte activation vial the IL-6 pathway, NF-κB mediates the systemic acute phase response and plays a central role in the initiation and maintaining of the vascular inflammation (Brasier, 2010; Perez et al., 2019).

IL-1β is another intriguing cytokine involved in the valve calcification process. Its levels are indeed increased in the stenotic aortic valve, making IL-1β subject to numerous research studies in the last decade (Isoda et al., 2010; Nadlonek et al., 2013; Lee and Choi, 2018). The IL-1β enhances the expression of matrix metalloproteinases (MMPs), a group of enzymes responsible for the degradation of extracellular matrix proteins. The MMPs exacerbate the process of valvular stenosis and activate the NF-κB pathway, leading to the production of several cytokines, such as IL-6, IL-8, and MCP-1 (monocyte chemoattractant protein-1) involved in both pathological processes, namely atherosclerosis and CAVD (Deshmane et al., 2009).

Finally, the IL-1 receptor agonist (IL-1Ra) plays a protective role in valvular disease, as its deficiency is closely associated with inflammatory cell infiltration and valve thickening (Isoda et al., 2010). Using *in vitro* and *in vivo* models, Zeng et al. elucidated the role of IL-37, an anti-inflammatory member of the IL-1 family. IL-37 attenuates the expression of bone morphogenetic protein 2 (BMP2) and alkaline phosphatase (ALP) enzyme, inhibiting the osteogenic process. In CAVD, the expression levels of IL-37 are very low, consequently, BMP2 is free to promote VICs calcification by ALP expression leading subsequently to aortic valvular thickening. Confirming the protective role of IL-37, *in vivo* experiments showed that mice expressing this human cytokine, displays significantly lower BMP-2 levels and a lower degree of aortic valve thickening (Zeng et al., 2017).

##### Adaptive Response in CAVD

The key role of lymphocytes in CAVD and the presence of T-lymphocyte clonal expansion in stenotic valves has been highlighted by various studies (Wallby et al., 2002; Mazzone et al., 2004; Mazur et al., 2018). In a study involving patients with severe CAVD, Wu et al. observed the presence of CD8 ^+^/CD28^−^ T cells near the mineralized nodules of the aortic valve and a higher prevalence of circulating CD3^+^ T cells, namely the subset of CD8^+^ and CD57^+^ T cells expressing HLA-DR, in subjects with CAVD (Wu et al., 2007). CD8^+^/CD28^−^ T cells play a crucial role in CAVD, as demonstrated by the correlation between the degree of clonal expansion and the severity of valve calcification. Furthermore, these cells play a crucial role in both activation and differentiation of memory effector status among circulating T cells. Winchester et al., investigated the composition of the lymphocytic infiltration in patients with (bi-and tri-cuspid CAVD). They showed an aggregate of infiltrating lymphocytes containing CD4^+^ and CD8^+^ T cells, with a preponderance of CD4 T cells. The clones shared between blood and the valve were found in the memory-effector CD8^+^CD28^−^ T cell subset, demonstrating the trafficking of members of the same T cell clone between the peripheral circulation and the valve. Importantly, the proportion of activation of peripheral blood T-lymphocytes strongly correlated with the degree of valve calcification, indicating that the extent of these events is somehow related to CAVD severity (Winchester et al., 2011).

This data suggests that in patients with CAVD, an adaptive systemic immune response is occurring, coupled with the valvular lymphocytic infiltration, probably triggered by recognition of antigens expressed in the valve. The valvular events that lead to an immune response, such as intracellular pathogens, or self-antigens, remain unclear. One hypothesis is that in response to shear stress the VICs express stress-induced molecules which could be perceived as antigens and generate an immune response.

#### Mineralization

At some point during the CAVD progression, VICs start to produce a calcified matrix and enter an osteogenic phase. The mechanisms of this switch are still poorly understood. One hypothetical pathway involves IL-6 and the tumor necrosis factor ligand superfamily member 11 (also called receptor activator of NF-κB ligand—RANKL). IL-6 has been shown to induce the expression of RANKL in bone cells, which activates its cognate receptor RANK that finally activates VICs to produce extracellular matrix and might therefore promote matrix calcification (Wada et al., 2006). This could be a hypothetical pathway, which through IL-6-RANKL overexpression promotes the osteogenic reprogramming of VICs (Kaden et al., 2004). Another pathway involving the expression of BMP2 *via* inflammatory cytokines and oxidized lipid derivatives was shown to induce osteogenic reprogramming in several cell types including VICs (Shao et al., 2005).

#### Genetic Factors Implicated in CAVD

Numerous genetic factors are implicated in the CAVD pathogenesis (LaHaye et al., 2014). Two recent genome-wide association (GWAS) studies showed that a genetic variation in the *LPA* locus, mediated by levels of Lp(a), is involved in both atherosclerotic disease and CAVD and that several pathways are shared by both conditions. Lp(a) and non-high-density lipoprotein cholesterol as shared risk factors contributed to the frequent co-existence of these disorders (Thanassoulis et al., 2013; Helgadottir et al., 2018).

A recent transcriptome-wide association study (TWAS) identified *PALMD* (palmdelphin), a gene that promotes myoblast differentiation and muscle regeneration, as an additional key gene in CAVD. The study showed that lowered expression levels of *PALMD* mRNA in valve tissues are associated with risk alleles for CAVD and higher disease severity (Nie et al., 2017; Theriault et al., 2018). Recently, Theriault et al. identified some additional susceptibility genes in patients with CAVD. Using both techniques, GWAS and TWAS, *IL-6*, *ALPL* (alkaline phosphatase), and *NAV1* (neuron navigator 1) emerged as important contributors involved not only in pulse and blood pressure modulation—two important factors associated with blood flow turbulence, already identified as risk factors for CAVD development—but also in the valve mineralization process. Moreover, the *ALPL* gene codes for tissue-nonspecific alkaline phosphatase, a crucial enzyme involved in mineralization. This gene was present in calcified aortic valves contrary to non-calcified valves (Thériault et al., 2019).

All these findings taken together constitute a new step toward the further elucidation of the CAVD pathogenesis and may open new paths for the development of innovative therapeutic approaches.

#### Gender as a Risk Factor for CAVD

The role of gender as a biological variable, especially that of the male sex as a risk factor for cardiovascular diseases, including CAVD, has been long established (Carroll et al., 1992; Institute of Medicine Board on Population H and Public Health P, 2012). However, data regarding the significance of male sex in CAVD is lacking. Indeed, most studies addressing sex-specific differences in CAVD focus on conditions caused by CAVD, such as ventricular dysfunction, rather than CAVD itself (Dobson et al., 2016). It is mostly unclear whether the onset and progression of CAVD are inherently different in men and women, due to genetic and cellular-scale sex differences, or whether the initial disease process is common in both sexes but undergoes subsequent differentiation after (Porras et al., 2017). Aggarwal et al. reported that male gender correlates with an higher incidence of CAVD, as men displayed higher levels of calcification for the same degree of aortic stenosis when compared to women (Aggarwal et al., 2013). Another study showed significant differences between stenotic valves in males and females, where the latter were more prone to fibrosis. (Sharma and Eghbali, 2014) Evidence points towards genetic and epigenetic differences, specifically the differential production of miRNAs, being the origin of these sex-specific differences (Sharma and Eghbali, 2014).

#### Epigenetic Regulation of CAVD

The remarkable developments in the field of epigenetics could offer answers regarding the link between environmental and proatherogenic factors, induction and perpetuation of inflammation, and CAVD development. The four major epigenetic mechanisms, collectively known as “epigenome”, include non-coding regulatory RNAs (ncRNA), DNA methylation, histone modifications, and chromatin remodeling (Murr, 2010). Studying the link between epigenome and CAVD could be clinically relevant since epigenetic markers could represent non-invasive biomarkers for monitoring CAVD initiation and progression and predicting prognosis.

ncRNAs, a group of single-stranded cellular RNAs made up of 18–25 nucleotides, represent a new area of interest in the field of human biology. These molecules, normally not coding for proteins, were previously regarded as “dark matter” since their function was largely unknown. Increasingly discovered to play a central role in the regulation of various molecular pathways and cell differentiation, ncRNAs are categorized in short, medium, or long (lncRNAs) based on their transcript size (Aryal et al., 2014). MicroRNAs (miRNAs) are the most well-studied ncRNAs, involved in cell development, proliferation, lipid metabolism, cancer metabolism, and angiogenesis (Palazzo and Lee, 2015). They regulate gene expression after transcription, by translational repression or transcript degradation. The role of miRNAs and especially lncRNAs in regulating atherosclerotic processes has been investigated in several studies suggesting that some miRNAs may be important inducers of proinflammatory pathways and others stimulators or inhibitors of calcification in the human aortic VICs (Gosev et al., 2017). Among others, miRNA-30b, miRNA-138, and miRNA-204 can suppress the differentiation of mesenchymal cells into osteoblasts whereas miRNA-29b and miRNA-214 promote calcification (Zhang et al., 2014; Wang et al., 2015; Fang et al., 2018; Lu et al., 2019; Zheng et al., 2019). miRNA-138 was indicated as a suppressor of osteoblastic differentiation of human aortic VICs by targeting FOXC1, representing, therefore, an inhibitor of VIC osteogenic differentiation during the development of CAVD (Lu et al., 2019). In 2017, the role of miRNA-29b as a promoter of valvular calcification, through the TGF-ß3/Smad3 and wnt3/ß-catenin pathways was reported (Fang et al., 2018) whereas in 2019, miRNA-214 was identified as a promoter of the human aortic VICs calcification by accelerating inflammatory chain reactions through MyD88/NF-κB signaling (Zheng et al., 2019).

Another epigenetic mark, the alteration of DNA methylation, could have a role in the osteogenic differentiation of aortic VICs by downregulating Notch/drosophila/homolog 1/translocation-associated (*NOTCH1*), a regulator of calcification-related gene networks in human valvular endothelium (Theodoris et al., 2015; Hadji et al., 2016). Markers of histone modification have also been reported to have a pro-inflammatory and osteogenic role in CAVD development. They participate in the shear-stress induced proinflammatory pathways *via* alteration of the silent information regulator-two (*SIRT)* gene expression (Roos et al., 2014).

In summary, increasing evidence supports the role of epigenetic factors as regulators of key processes underlying valvular tissue remodeling and participators in the landscape of phenotypical changes occurring in CAVD. Understanding the epigenetic mechanisms involved in the initiation and progression of CAVD will not only help to enlighten the pathology, but since these markers are potentially reversible, it could offer important targets for diagnostic and therapeutic interventions.

## The Putative Role of the Microbiome

Herein we hypothesize a putative role of the microbiome in the development and progression of CAVD, either *via* stimulation of immune response and valve calcification or *via* direct valvular damage caused by specific bacterial taxa. Different hints point to a role microbiome in the promotion of CAVD and its deterioration to severe calcified aortic stenosis. Some known risk factors for CAVD, such as age, male sex, hypertension, and diabetes mellitus, have a known relationship to the microbiome. In fact, patients with type II diabetes mellitus (T2DM), which is associated with the gut microbiota dysbiosis, are at higher risk for developing CAVD (Harsch and Konturek, 2018). Tissue histopathological studies demonstrated more calcification in tissue samples from patients with T2DM compared to non-diabetic patients (Mosch et al., 2017). Furthermore, the presence of metabolic syndrome is associated with faster disease progression and worse outcome in patients with aortic stenosis (Katz et al., 2009). Here we discuss in detail the involvement of oral and gut microbiome (GM) in pathophysiological processes that promote cardiovascular diseases, including CAVD.

### Oral Microbiome

The oral microbiome represents, after the GM, the second-largest microbial community in humans and plays a fundamental role in maintaining the oral cavity homeostasis and preventing the development of several diseases (Jia et al., 2018; Deo and Deshmukh, 2019). The involvement of the oral microbiome in pathologies such as obesity, diabetes, cancer, and cardiovascular diseases is well documented (Gao et al., 2016; Eberhard et al., 2017; Long et al., 2017; Yang et al., 2019). Different studies suggest that oral pathologies with oral microbiome alteration, have a tight link with heart diseases; for example, the gingival ulceration in periodontitis causes bacteremia and could induce the formation of atherosclerotic plaques (DeStefano et al., 1993; Bartova et al., 2014). Increasing evidence suggests a close association between oral microbiome alteration and CAVD, demonstrating the presence of oral bacteria in the valvular tissue by using a polymerase chain reaction (PCR) (Nakano et al., 2006). Nomura et al., investigated the mechanisms through which *Streptococcus mutans* (*S. mutants*), the major pathogen responsible for dental caries, colonizes heart valves constituting a potential virulence factor for the development of infective endocarditis. They demonstrated that specific strains of *S. mutants* have selective virulence for infectious endocarditis (Nomura et al., 2014; Oliveira et al., 2015). There is an increasingly accepted hypothesis that bacterial endocarditis is one of the causes of aortic valve calcification, supported by *in vivo* studies showing larger calcifications in animals inoculated with endocarditis-related pathogens.(Cohen et al., 2004)

### Gut Microbiome

The composition of the human microbiome is specific to each individual and the study of the GM is increasingly focusing on its role in the physiology of the host organism in during phases of health and disease (Blander et al., 2017). Changes in the composition of GM are involved in several diseases including atherosclerosis, hypertension, heart failure, chronic kidney disease, obesity, diabetes, inflammatory bowel disease, and colon cancer (Amedei and Barcelo-Coblijn, 2019). Growing evidence suggests that intestinal bacteria play an essential role in cardiovascular diseases. GM metabolites such as choline, betaine, and trimethylamine N-oxide (TMAO), are the subject of research studies investigating the correlation between the intestinal microbiome and the calcification of the aortic valve. Diets based on red meat, eggs, and dairy products are rich in choline, lecithin, and carnitine, constituting, therefore, a potential source of TMAO. Recent metabolomic studies indicate that fasting TMAO, choline, and phosphatidylcholine plasma levels are related to high risk of cardiovascular diseases. Furthermore, carnitine has been indicated as an independent predictor of major adverse cardiovascular events, with TMAO as the main driver behind the risk association (Velasquez et al., 2016; Canyelles et al., 2018). The idea that gut microbiota could contribute to CAVD is new. Very recently Kocyigit et al. reported a significant association between gut microbiota metabolites and CAVD (Kocyigit et al., 2017) whereas Liu et al. showed that patients with CAVD and those with coronary artery disease suffer from different gut microbial dysbiosis (Liu et al., 2019). The next section discusses the putative link between the GM and CAVD development.

### The Role of the GM in Adaptive Immunity

Several studies indicate a very close connection between the GM and adaptive immunity. Bacterial metabolites function as a link between the commensal microbiome and the immune system, by affecting the shifting of the immune response between pro- and anti-inflammatory pathways. Hypothetically, the gap between the microbiome and immune system could be filled by short-chain fatty acids (SCFAs), generated by bacterial fermentation of dietary fiber in the intestinal lumen (Russo et al., 2016). SCFAs such as acetate, propionate, and butyrate act as important inductors of regulatory T (Treg) cell differentiation and reinforce the gut barrier function by expressing the transcription factor Foxp3 and consequently suppressing inflammatory responses in the intestine (Arpaia et al., 2013). A diet rich in SCFAs might antagonize several immunological defects, therefore GM-derived metabolites could offer a therapeutic advantage for patients suffering from various immunological conditions (Luu et al., 2019).

Significant links between fungal- and bacterial-induced cytokine responses and specific gut bacterial species were identified through the Human Functional Genomics Project. For instance, the production of interferon-γ (IFN-γ) and TNF-α is strongly associated with the GM (Schirmer et al., 2016). The fermentation of bacterial SCFAs acts through activation of G-protein coupled receptors by modulating the activity of intestinal epithelial cells and leukocyte life cycle. Furthermore, due to their direct effect on lymphocytes and by activating macrophages and dendric cells, SCFAs can induce a T-lymphocyte tolerogenic profile, acting as a link between the microbiome and the immune system (Correa-Oliveira et al., 2016).

**Figure 3:**
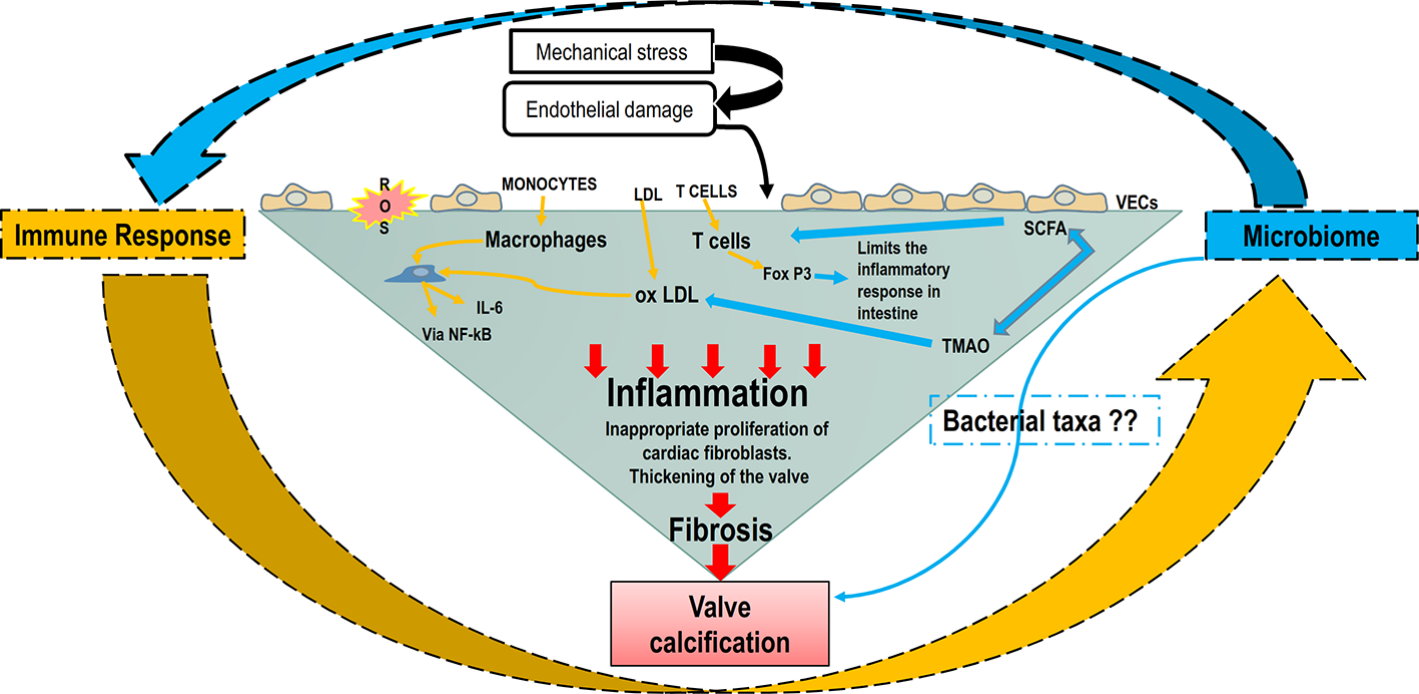
The putative interaction between the microbiome and immune response in CAVD development. Increased shear stress, genetic factors, and oxygen reactive species cause the endothelial damage that allows ox-LDLs, immune and inflammatory cells to infiltrate the valve. Macrophages and ox-LDLs stimulate VICs inflammation through NF-κB/IL-6 signaling pathway. The microbiome and microbial metabolites can modulate the immune system through the intense effect of SFCAs on T cells’ differentiation. Furthermore, the bacterial metabolite TMAO can induce ox-LDL and maintain the inflammatory loop. IL-6, interleukin 6; NF-κB, nuclear factor κ-light-chain enhancer of activated B cells; ox-LDL, oxidized low-density lipoprotein; SCFA, short-chain fatty acid; TMAO-Trimethylamine-N oxide; VECs, valve endothelial cells; VICs, vascular interstitial cells.

In summary, the role of adaptive immunity in CAVD has been explored, but the valvular events that lead to an immune response, such as intracellular pathogens or self-antigens, remain largely undetermined. Alike atherosclerosis, one could hypothesize that the presence of a certain type of gut or mouth microbiome could lead to the presence of bacterial DNA inducing valvular specific antigens that could trigger an immune response. The putative interaction between the microbiome and immune response leading to CAVD development is schematized in **Figure 3**.

## Novel Potential Therapeutic Strategies

Currently, no drug strategies can prevent or reverse CAVD and valve replacement remains the only treatment option. Patients with severe CAVD undergo valve replacement either with surgery or *via* percutaneous transcatheter procedures. Drugs already used to treat vascular complications might also improve CAVD outcomes, but the mechanisms of valve calcification differ from other vascular conditions (Hutcheson et al., 2014).

A randomized trial of intensive lipid-lowering therapy failed to stop ore reverse the progression of CAVD. However, the possibility that this specific pharmacological treatment induces a small reduction in disease progression or in the main clinical endpoints cannot be excluded (Cowell et al., 2005; Rossebo et al., 2008). Anti-inflammatory agents represent a promising therapeutic strategy that requires additional research in CAVD patients (Hurle et al., 2013). Due to the role of angiotensin II in CAVD development, inhibitors of angiotensin-converting enzyme (ACE-I) have also been explored. Notably, no significant difference in disease progression was observed in a retrospective cohort of more than 200 patients. The benefits of ACE-I are mostly related to their effect on left ventricular remodeling.

Human monoclonal antibodies have been used successfully in many conditions. In 2015 Lerman et al. found that denosumab, a human monoclonal antibody targeting the receptor activator of nuclear factor-κB ligand, may reduce valvular calcium deposition to basal levels (Lerman et al., 2015). Furthermore, denosumab reduced calcium deposition in the aorta, although the mechanisms by which it affected ectopic calcification, must be further examined. (Helas et al., 2009) Nonetheless, this field remains attractive and promising.

After GWAS and TWAS studies discovering several genes implicated in the CAVD pathogenesis, genetic therapy could also be a possible therapeutic development. A growing body of experimental evidence supports the role of epigenetic factors such as miRNAs and markers of DNA methylation, as regulators of key processes underlying valvular tissue remodeling and participators in the landscape of phenotypical changes occurring in CAVD. Since these markers are potentially reversible, they could offer important targets for diagnostic and therapeutic interventions.

The recently introduced, tissue-engineered heart valves [tissue engineered heart valves (TEHVs)] may constitute a valid alternative to traditional strategies of valve replacement. In TEHVs, a decellularized scaffold is seeded with patient’s cells subjected to an appropriate series of stimuli able to promote cellular activity. TEHVs could offer the advantages of biocompatibility and longevity, while preventing pathological tissue responses and regurgitation. Additionally, TEHVs could allow VICs to produce their own extracellular matrix, thus displaying the capacity for growth and remodeling, which is critical for pediatric patients. TEHVs can be produced from synthetic or natural materials (Berry et al., 2010).

## Conclusions

Despite its high prevalence and associated mortality, there are no drugs to prevent or cure CAVD. Therefore, the development of new therapeutic strategies capable of counteracting the CAVD development is crucial.

Being a multifactorial disease, CAVD is characterized by a complex pathobiology that involves environmental and genetic factors, immune-molecular pathways, hemodynamic factors, and shear stress, all intertwined in the systemic district. Several of the many steps of disease progression have been clarified but some others, like the critical switch from inflammatory-fibrotic to osteogenic program, or the putative role of microbiota, remain still unclear. The quickly evolving field of epigenetic regulation of CAVD, involving especially miRNAs and lncRNAs, could offer novel potential biomarkers for the development of new diagnostic and therapeutic interventions. Furthermore, the microbiome could play a role in promoting and perpetuating CAVD by inducing the production of endogenous or exogenous antigens *via* the activation of inflammatory pathways. The elucidation of these conundrums will help identify new disease targets, host or microbial, and support the design of new potential therapeutic strategies to prevent or reverse the effects of CAVD.

## Author Contributions

BA, LC, AL, and AA were involved in the study concept and design. BA, LC, and MC drafted the manuscript. AL, UL, HG, and AA revised the manuscript for important intellectual contents. All authors had access to all the data, including figures, and approved the manuscript for final submission.

## Conflict of Interest

The authors declare that the research was conducted in the absence of any commercial or financial relationships that could be construed as a potential conflict of interest.
